# The great Indian joint families of free-ranging dogs

**DOI:** 10.1371/journal.pone.0197328

**Published:** 2018-05-17

**Authors:** Manabi Paul, Anindita Bhadra

**Affiliations:** Behaviour and Ecology Lab, Department of Biological Sciences, Indian Institute of Science Education and Research Kolkata, Mohanpur, India; Institute of Animal Science, CZECH REPUBLIC

## Abstract

Cooperative breeding is an excellent example of cooperation in social groups. Domestic dogs have evolved from cooperatively hunting and breeding ancestors but have adapted to a facultatively social scavenging lifestyle on streets, and solitary living in human homes. Pets typically breed and reproduce under human supervision, but free-ranging dogs can provide insights into the natural breeding ecology of dogs. We conducted a five year-long field based behavioural study on parental care of free-ranging dogs in India. 23 mother-litter units, belonging to 15 groups were observed, which revealed the presence of widespread allo-parenting by both adult males and females. While all the females were known to be related to the pups receiving care, the relatedness with the males could not be determined. Hence, we coined the term “putative father” for caregiving males. Allomothers provided significantly less care than the mothers, but the putative fathers showed comparable levels of care with the mothers. Mothers invested more effort in nursing and allogrooming, while the putative fathers played and protected more. Our observations provide support for both the “benefit-of-philopatry” and “assured fitness returns” hypotheses. Free-ranging dogs are not cooperative breeders like wolves but are rather communal breeders; their breeding biology bearing interesting similarities with the human joint family system. This breeding strategy is likely to have played an important role in increasing pup survival in a stochastic environment and helping to adapt to living among humans during the domestication of dogs.

## Introduction

Cooperation and conflict drive the dynamics of social groups in species as diverse as insects to humans. While selfishness is easily explained by the theory of natural selection [1,2], cooperation between individuals, sometimes at the cost of fitness, and altruism, where an individual sacrifices its fitness for the benefit of another, are more difficult to understand as behavioural traits [3]. An extreme form of cooperation in animal societies is manifested by cooperative breeding, where only a few individuals in a social group reproduce and the others help to rear their offspring, forfeiting reproduction themselves [4,5]. Cooperative breeding is commonly observed in social insects, and in some birds, but is relatively less common in mammals. Mammals, for example, naked mole rats, lemurs, meerkats and some species of canids including wolves and coyotes are cooperative breeders [5–9]. Cooperative breeding is sometimes confused with communal breeding, though these are quite distinct breeding systems. In communally breeding species, multiple females share dens or birthing sites and help each other to rear offspring, but do not forfeit their opportunities to reproduce [6,8]. Thus species cooperating in breeding usually vary in the degree to which dominants suppress the reproduction of subordinates [10,11].

Kin selection theory suggests that individuals can maximize inclusive fitness by helping genetic relatives, and thus apparently altruistic behaviours can evolve in species where closely related individuals tend to stay together, which in turn can lead to the evolution of eusociality [12,13]. Though the advantage of cooperative breeding has mostly been explained by kin selection theory it has been argued that the benefits gained by the alloparent through inclusive fitness are not enough to compensate for their own reproduction [14]. Philopatry, or the tendency of adults to remain in the natal areas, leads to an increased probability of kin living in close proximity, thereby facilitating kin selection by elevating the relatedness between the care giver and recipient. Hence, for philopatric species, altruistic behaviours like providing care to non-filial offspring can indeed provide inclusive fitness benefits through kin selection [12,13]. Females of most group-living mammals are reported to be philopatric [15] and the average kinship between females is highest for smaller groups [16,17]. Hence cooperative breeding could be a consequence of kin selection induced by philopatry in such groups; for example, subordinates of a wolf pack are usually philopatric offspring of the dominant breeding pair [18,19]. The occurrence of communal breeding, on the other hand, does not demand an explanation based on genetic advantages, but can simply enhance fitness of individuals due to the adults providing some additional care to each other’s offspring, leading to advantages for the group [20].

Domestic dogs [*Canis lupus familiaris*] share a common ancestry with modern gray wolves [*Canis lupus lupus*] [21], but show much variation in their social organization. They are capable of living solitarily as pets, in groups artificially assembled by humans, and as natural social groups in free-ranging populations [22–24]. Free-ranging dogs comprise of nearly 70–80% of the world’s dog population [25,26], and represent a condition in which dogs exist as naturally breeding populations, independent of direct human supervision [22]. Studying such populations of dogs can provide interesting insights into the ecology and ethology of dogs, and help us to understand how ancestral dogs adapted to humans, eventually becoming the first animal to have been domesticated.

Free-ranging dogs in India have a promiscuous mating system, with multiple males and females mating within a group in a given season [27,28]. Their group dynamics greatly depend on their mating and denning seasons [24], and often multiple females of a group give birth in neighbouring dens. Mothers provide extensive care to the developing pups [29] but adjust the levels of care with pup age and litter sizes [30]. They are predominantly scavengers, surviving on human-generated waste, but are capable of forming large packs to hunt down animals like goats and deer [31–33]. Free-ranging dogs do not have reproductive hierarchies like cooperatively breeding canids, but do show allocare by males and related females to some extent [29,34]. In a case study we reported allocare by a non-lactating grandmother towards her grandpups in the presence of the mother [34]. Milk-theft is also observed in many groups of free-ranging dogs where multiple females give birth in the same breeding season, and pups tend to opportunistically suckle from any available lactating female in the group [35]. Free-ranging dogs are capable of forming large packs, in which social hierarchies are evident, which also influence the reproductive behaviour of the adults, in spite of the overall mating system being promiscuous [36]. Thus, the free-ranging dogs show a large degree of flexibility in their social behaviour and present an interesting situation which seems to be somewhat intermediate between the strict social hierarchies observed in wolves, coyotes, wild dogs, etc. and the solitary lifestyle of canids like foxes and jackals. There has never been a comparative assessment of the various forms of care received by developing pups, which is important to understand the breeding biology of dogs. We carried out a long term behavioural study in India to understand the nature of alloparenting behaviour in groups of free-ranging dogs. We hypothesized that the primary care received by pups would be from the mothers, and allocare by both males and females would be present at a lower level than maternal care. We expected allocare to be mostly present in the form of passive care, as this can ensure social cohesion within the group at little cost to the caregiver.

## Materials and methods

We carried out field-based observations to collect behavioural data from 15 dog groups in West Bengal, India, over a span of four denning seasons (2010–11, 2011–12, 2013–14 and 2014–15). The study sites were in urban and suburban residential areas that were selected on the basis of availability of dog groups and convenience of long term observations. A group was defined by individuals that defended a common territory and shared resources like food and shelter, prior to the denning season. Each dog group consisted of one or more adults and pups/ juveniles. All individuals in the group were uniquely named according to their coat colour and patch patterns. A group could have more than one lactating female with her current litter. We used the litters as our focal groups for behavioural observations and recorded all behavioural interactions initiated and received by them. Thus, we collected data on 23 litters (having a total of 84 pups and 50 adults including 23 lactating females) belonging to 15 dog groups. Each litter was followed over a period of 15 weeks, from the 3^rd^ to 17^th^ weeks of pup age, and observed for two morning (0900-1200h) and two evening (1400-1700h) sessions spread over two-week blocks. Each three hour observation session consisted of 18 instantaneous scans and 18 all occurrences sessions (AOS), amounting to a total of 12420 scans of one minute each and 12420 AOS of five minutes each [37].

Any behaviour that was shown by an adult towards a pup, which was expected to enhance the pup’s chances of survival, was designated as care [38]. We maintained the birth details of focal individuals for the last five years (2010–2015), which enable us to know the relationships between individuals through the mother. However, we did not have genetic data for calculating relatedness between individuals, as we were not sure of the paternity of the pups. We had qualitative observations on mating within the groups and knew which males each female had mated with. Thus, any adult male that provided care in any form to the pups was designated as a “putative father” (PF). Any adult female other than the mother (MO) who provided care to the pups was designated as an allomother (AM). One of the litters (PF_2_) lost their mother in a car accident a day after their birth, and their grandmother (maternal) “adopted” them and took care of them till her death in the 10^th^ week of their age. See Table 1 for the group details. Care shown by the mother, putative father and allomother were labeled as maternal care, male care and female-allocare respectively. We sorted the total care shown by the adults towards the focal pups into two categories, active care and passive care. Any behaviour that involved direct interaction between the care giver and the pups, and was energy intensive (nursing, allogrooming, food provisioning, play, pile sleep, direct protection by chasing or fighting with strangers, etc.) was considered as active care. Behaviours that require no direct interaction with the focal pups, but can provide care in terms of indirect protection and social bonding due to the adult’s presence in the pups’ vicinity were considered as passive care [30,35]. See S1 Table for the detailed ethogram.

**Table 1:** Details of observed dog groups.

Sl no.	Year	Group name	Litter id	LS	Group size	Group composition	No. of AMs	Relationship between pups and AM
1	2010–11	CAN1	CAN1	5	9	MO + P (5)+ AM (0) + PF (0) + OJ (0) + OD (1F, 2M)	-	-
2	2010–11	BUD	BUD1	4	6	MO + P (4) + AM (0) + PF (0) + OJ (0) + OD (1M)	-	-
3	2010–11	LEL1	LEL1	2	3	MO + P (2) + AM (0) + PF (0) + OJ (0) + OD (0)	-	-
4	2010–11	S1	S1	2	3	MO + P (2) + AM (0) + PF (0) + OJ (0) + OD (0)	-	-
5	2011–12	BSF1	RS4	5	11	MO + P (5) + AM (0) + PF (1) + OJ (2) + OD (1F, 1M)	-	-
6	2011–12	PLT1	JCB	2	9	MO + P (2) + AM (1) + PF (0) + OJ (5) + OD (0)	1	Elder sister (r = 0.25)
7	2011–12	PLT1	MDB1	5	10	MO + P (5) + AM (1) + PF (1) + OJ (2) + OD (0)	1	Grandmother (r = 0.25)
8	2011–12	CAN2	CAN2	5	9	MO + P (5) + AM (1) + PF (0) + OJ (0) + OD (2M)	1	Grandmother (r = 0.25)
9	2011–12	GH	GH2	6	8	MO + P (6) + AM (1) + PF (0) + OJ (0) + OD (0)	1	Aunt (r = 0.125)
10	2011–12	LEL2	LEL2	2	5	MO + P (2) + AM (1) + PF (0) + OJ (0) + OD (1M)	1	Grandmother (r = 0.25)
11	2011–12	S2	S2	3	7	MO + P (3) + AM (0) + PF (1) + OJ (0) + OD (2M)	-	-
12	2013–14	BSF2	RS1	2	12	MO + P (2) + AM (1) + PF (1) + OJ (6) + OD (1F)	1	Elder sister (r = 0.25)
13	2013–14	BSF2	RS2	4	12	MO + P (4) + AM (1) + PF (1) + OJ (4)+ OD (1F)	1	Aunt (r = 0.125)
14	2013–14	BSF2	RS3	2	12	MO + P (2) + AM (1) + PF (1) + OJ (6)+ OD (1F)	1	Aunt (r = 0.125)
15	2013–14	PF	PF1	5	9	MO + P (5) + AM (1) + PF (1) + OJ (0) + OD (1F)	1	Unknown
16	2013–14	PF	**PF**_**2**_	6	9	**P (6) + AM (2) + PF (1) + OJ (0)** + OD (0)	2	Grandmother (r = 0.25); aunt (r = 0.125).
17	2013–14	CAN3	CAN3	6	8	MO + P (6) + AM (0) + PF (1) + OJ (0) + OD (0)	-	-
18	2013–14	PLT2	MDB2	5	8	MO + P (5) + AM (0) + PF (1) + OJ (1) + OD (0)	-	-
19	2014–15	BSF3	BBR	4	20	MO + P (4) + AM (4) + PF (1) + OJ (9)+ OD (1F)	4	Elder sister (r = 0.25); aunt (r = 0.125); grandmother (r = 0.25); cousin (r = 0.0625).
20	2014–15	BSF3	KTI	2	20	MO + P (2) + AM (4) + PF (1) + OJ (11)+ OD (1F)	4	Aunt (r = 0.125); grandmother (r = 0.25); Mother’s aunt and Mother’s grandmother (r = 0.125).
21	2014–15	BSF3	WHI	2	20	MO + P (2) + AM (4) + PF (1) + OJ (11)+ OD (1F)	4	Mother’s niece and aunt (r = 0.125); grandmother (r = 0.25); cousin (r = 0.625).
22	2014–15	BSF3	BRN	2	20	MO + P (2) + AM (4) + PF (1) + OJ (11)+ OD (1F)	4	Aunt (r = 0.125); grandmother (r = 0.25); Mother’s aunt and Mother’s grandmother (r = 0.125).
23	2014–15	BSF3	RS5	3	20	MO + P (3) + AM (4) + PF (1) + OJ (10)+ OD (1F)	4	2 elder sisters (r = 0.25); 2 nieces (r = 0.125).

Table showing the details of 23 litters from 15 dog groups, including the minimum relatedness (r) between pups (P) and allomothers (considering random mating with unrelated males]. MO, PF, AM and OD have been used as the codes for the mother, putative fathers, allomothers and other adult dogs in the group, respectively. Dogs that were present in the groups but did not show any form of care to the pups were labelled as OD. OJ represents the pups/juveniles, other than the focal pups. PF_2_ group lost their mother and received care from AM and PF.

Since the pups of PF_2_ received grandmother’s care instead of their mother’s, we have considered this group as a special case and compared them with both the maternal and allomaternal care. Nursing is considered as the most energetically demanding form of active care that the mother provides to her pups. There is a scope for the grandmother to provide equal levels of care as the mother by investing less in nursing and more in other active care behaviours, and ultimately investing less energy in providing care to the adopted pups. Hence, we analyzed the grandmother’s care both for the total active care (that includes nursing) and nursing only.

### Statistics

We used generalized linear mixed models (GLMM) to test the effect of pup age and litter size on the care given by the PF and the AM separately. The total number of scans were sorted into four categories such as active care, passive care, inactive (sleeping or lazing) and absent (PF or AM was absent during the scan). The total number of scans varied for the different groups and weeks as it depended on the availability of the focal individuals and weather conditions. Hence, we used the “proportion of time spent in active care” and “proportion of time spent in passive care” as the response variables for both PF and AM. Since we collected data from 23 litters, over a span of four years, we incorporated the litter identities (ID) and the year of data collection (Yr) as the “random effects” in GLMM. We used the function “lme” from the R package “nlme” to incorporate both the “fixed effects” and “random effects” in the GLMM [39,40]. We used “fitdistrplus” package [41] to check the best fit distribution of the data set and compared the AIC values; AIC value was lowest for the “normal distribution” (S2 Table). Thus, a Gaussian distribution was considered for the response variable with a linear linking function (link = “identity”) in the GLMM. Pup age (in weeks) and litter size (ls) was incorporated as the “fixed effects”. Since the litter size changed over weeks due to pup mortality, we considered the current litter size in each week for the analysis. We used the Bartlett’s test of homogeneity of variances to check the presence of homoscedasticity, separately for two predictor variables i.e. age and litter size. We ran two GLMMs, one showing the effect of pup age and litter size on the proportion of time spent in active care and the other on passive care, each for PF and AM.

We considered the proportion of time invested in care (either active or passive) as the response variable and the caregivers (MO, PF, and AM who provided the care) as the categorical predictor factor for ANOVA.

### Inter-observer variability

Two observers were involved in carrying out the behavioural observations. Before commencing the study, pilot observations were conducted simultaneously by the two observers on a group of dogs, and their records were tested for inter-observer variability. This was done for three dog groups, and for 10 hours of data. Since inter-observer variability was not found to be significant, the two observers continued with the study.

We used StatistiXL 1.10, Statistica version 12 and R statistics for the statistical analysis.

### Ethics statements

No dogs were harmed during this work. All work reported here was purely observation based and did not involve handling of dogs in any manner. The methods reported in this paper were approved by the animal ethics committee of IISER Kolkata (approval number: 1385/ac/10/CPCSEA) and was in accordance with approved guidelines of animal rights regulations of the Government of India.

## Results and discussions

Allocare was observed in 19 out of the 23 litters, of which 10 litters received all the three types of care i.e. maternal care, male care and female-allocare. Four litters received male care and maternal care while four others received female-allocare and maternal care. Thus, male care was observed in 14 litters and female-allocare in 14 litters. Pups of PF_2_ group received only allocare from their grandmother and putative father (Table 1, S1 Fig).

### (i) Active care

**Fig 1:**
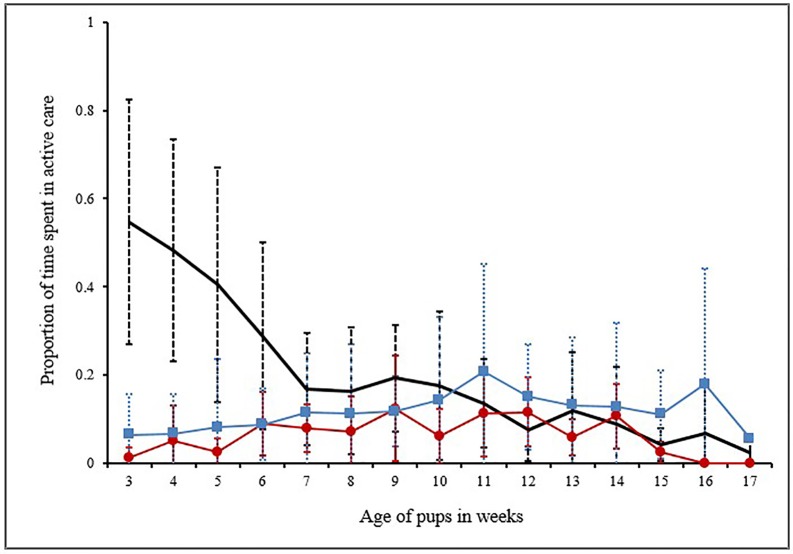
Graphical representation for proportion of time spent in active care. Line graph showing the mean and standard deviation of proportion of time spent in active care by the MO (n = 22), PF (n = 14) and AM (n = 14). The thick line designates the MO, thin line with solid squares designates PF and the line with solid circles is for AM.

The levels of active care shown by the mother varied with the age of pups (Mean ± SD: 19.85 ± 16.12%) out of the total time of observations, with the highest care (54.69%) being shown in the 3^rd^ week of pup age. Contrary to our expectations, active allocare was observed to be provided by both males and females. Unlike maternal care which depends on both litter size and the age of the pups [30], active allocare by both males and females depended only on pup age (see GLMMs in Table 2, S1 Text). Mean ± SD of active male care was (11.69 ± 4.23)%, with the highest active care being shown in the 11^th^ week (21%). In case of active female allocare was (6.54 ± 4.1)%, the highest care was shown in the 9^th^ week (11.8%). The levels of active care shown by MO, AM and PF varied significantly (ANOVA: *F*_*2*,*42*_ = 6.89, *P* = 0.003). Active care by the AM was significantly lower than active maternal care (Post hoc Tukey test: *P* = 0.002) (Fig 1), which was not surprising. However, active care shown by PF was comparable to the levels of active care shown by the mother (Post hoc Tukey test: *P* = 0.07) (Fig 1). We compared male and female allocare separately with maternal care in order to analyze the underlying differences in the patterns of care.

**Table 2:** The results of the GLMM for active cares shown by the PF and AM.

	Value	Std. Error	t-value	p-value
**Putative father (PF)**				
(Intercept)	0.04	0.05	0.65	0.52
age	0.006	0.002	2.21	0.03*
ls	0.005	0.01	0.5	0.62
**Allomother (AM)**				
(Intercept)	0.04	0.04	1.09	0.07
age	0.003	0.001	2.07	0.04*
ls	0.001	0.005	0.01	0.9

The results of the GLMM considering the proportion of time spent in active care by the PF and AM as the response variable. Pup age (age) in weeks and litter size (ls) were incorporated as the fixed effects. Litter size ranged from 2 to 6, and the age of pups ranged from 3 to 17 weeks. The identities of 14 litters (gr) separately for PF and AM and the year of data collection (yr) were the “random effects”.

(*) depicts significant effect.

#### (a) Maternal care vs male care

**Fig 2:**
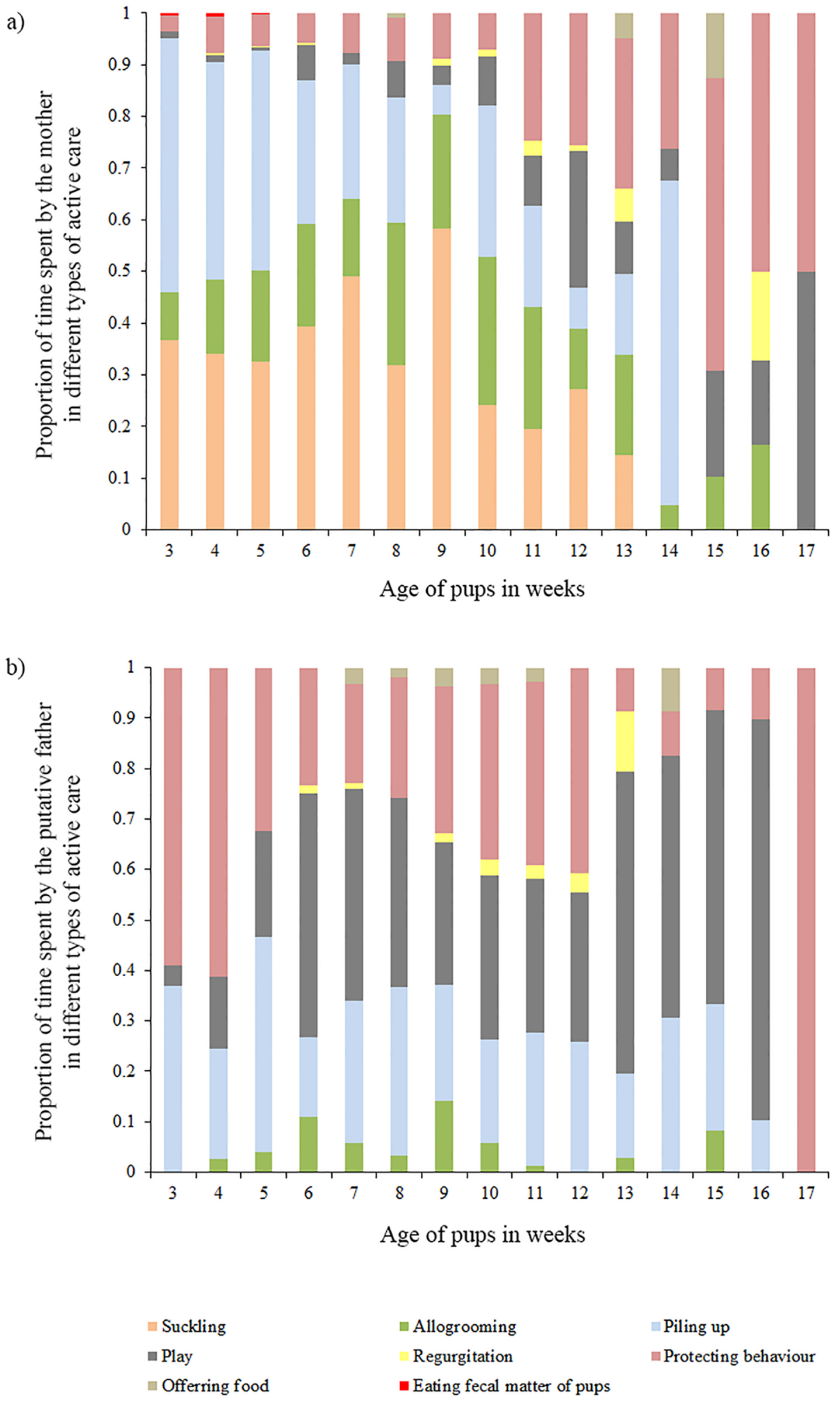
Time activity budget of mothers and putative fathers. a) Stacked bar diagram showing how the mothers budgeted their time in the various care-giving behaviours over pup age. b) Stacked bar diagram showing the proportion of time spent by the putative fathers in various active care over pup age.

Mothers showed significantly higher levels of active care than the PF in the pre-weaning phase of pup development (3^rd^ to 7^th^ week), when the pups were being voluntarily nursed by the mother (2 tailed T test: *T* = 3.82, *DF* = 5, *P* = 0.01) (Fig 1). Moreover, throughout the observation period (3^rd^ to 17^th^ week) MO and PF budgeted their time in the various care-giving behaviours differently (Contingency Chi Sq. test: *χ2* = 48.2, *DF* = 7, *P*< 0.0001). For the first three weeks of observations (3^rd^ to 5^th^ week of pup age), 76% to 86% of the active maternal care comprised of nursing and pile sleeping. Play and protection replaced these behaviours as the pups grew older (Fig 2a). In case of male care, play and protection consistently contributed to 69 ± 12% of active male-allocare, throughout the entire period of observations (Fig 2b).

#### (b) Maternal care vs female-allocare

**Fig 3:**
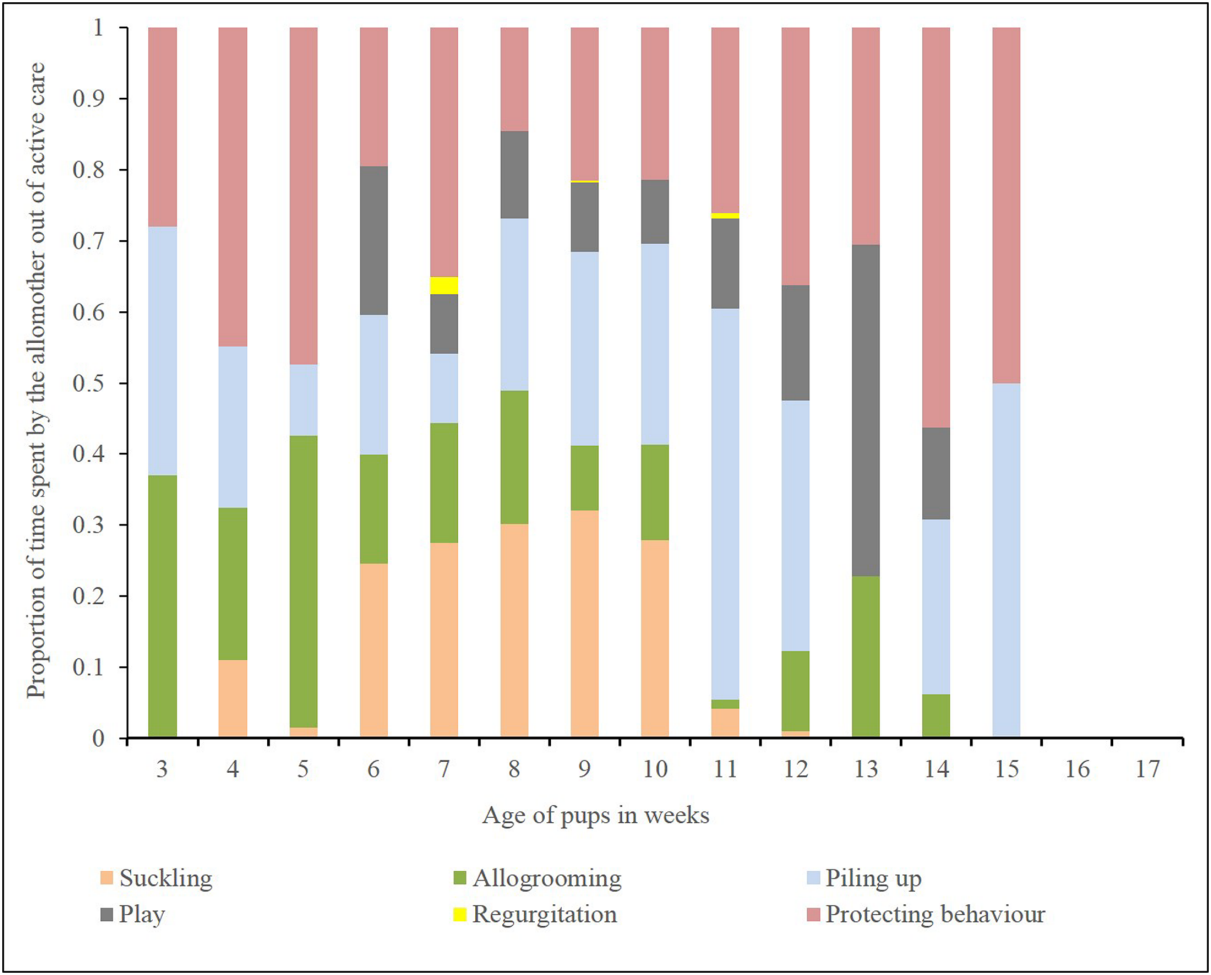
Time activity budget of allomothers. Stacked bar diagram showing the proportion of time spent by the allomothers in various active care behaviours over pup age.

**Fig 4:**
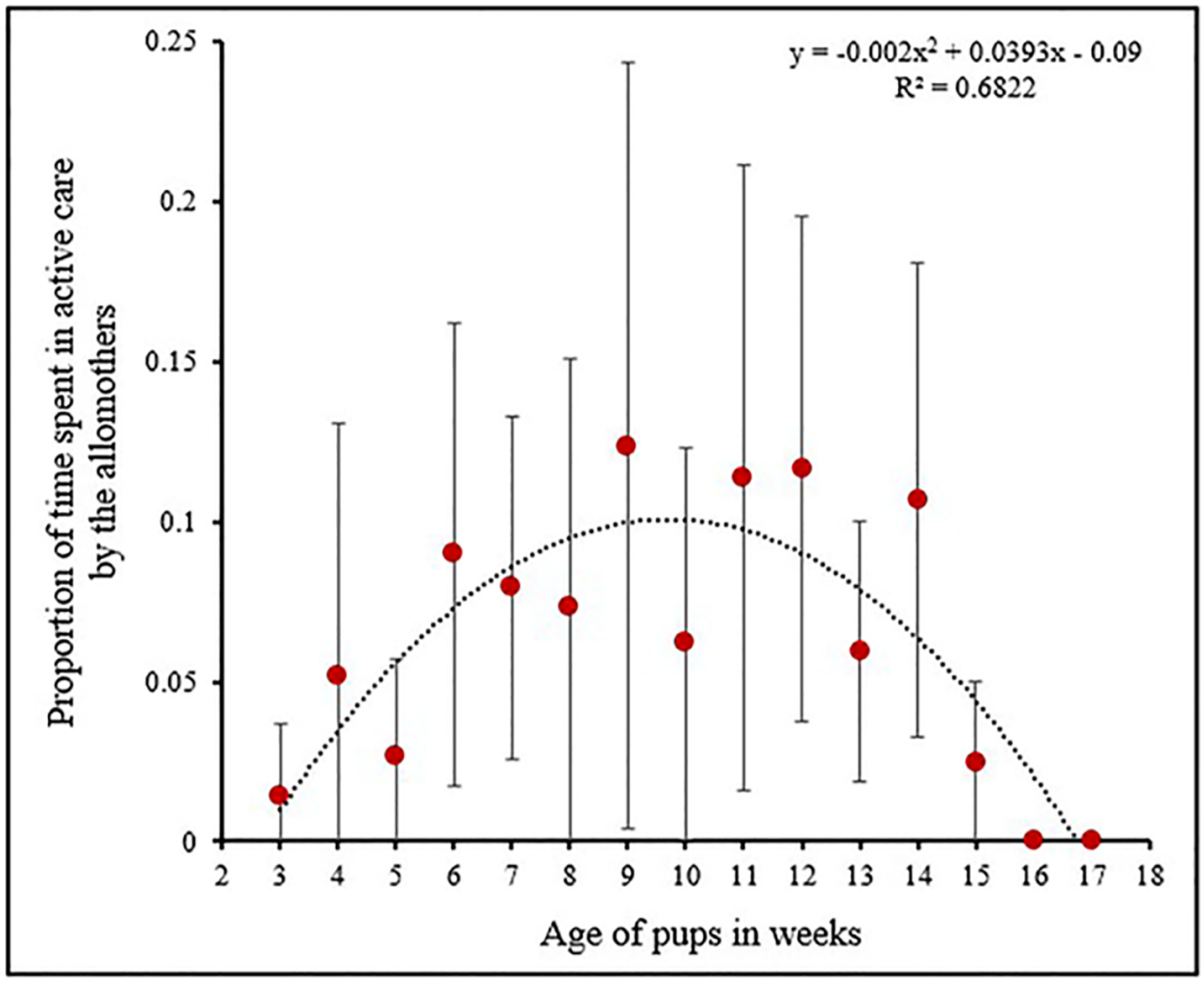
Patterns of active female allocare. Mean values of the proportion of time spent in active care by the AM at different pup ages, with a quadratic function fit. The function peaks between 9^th^ to 10^th^ weeks of pup age.

Pups received female-allocare as early as their 3^rd^ week of age, but the level of female-allocare was significantly lower than maternal care (2 tailed T test: *T* = 3.15, *DF* = 14, *P* = 0.006) (Fig 1). Over weeks allomothers budgeted their time in the various care-giving behaviours differently (Fig 3). The level of female-allocare increased from the 3^rd^ week of pup age and reached its peak between 9^th^ and 10^th^ week of pup age, decreasing again as the pups grew older (Quadratic regression: *R*^*2*^ = 0.68, *F* = 12.88, *DF* = 2, 12, *P* = 0.001, S2 Table) (Fig 4). All the AM were related to the pups to which they provided care. Here we present the relatedness estimates based on birth records, considering random mating with unrelated males (Table 1).

### (ii) Passive care

**Fig 5:**
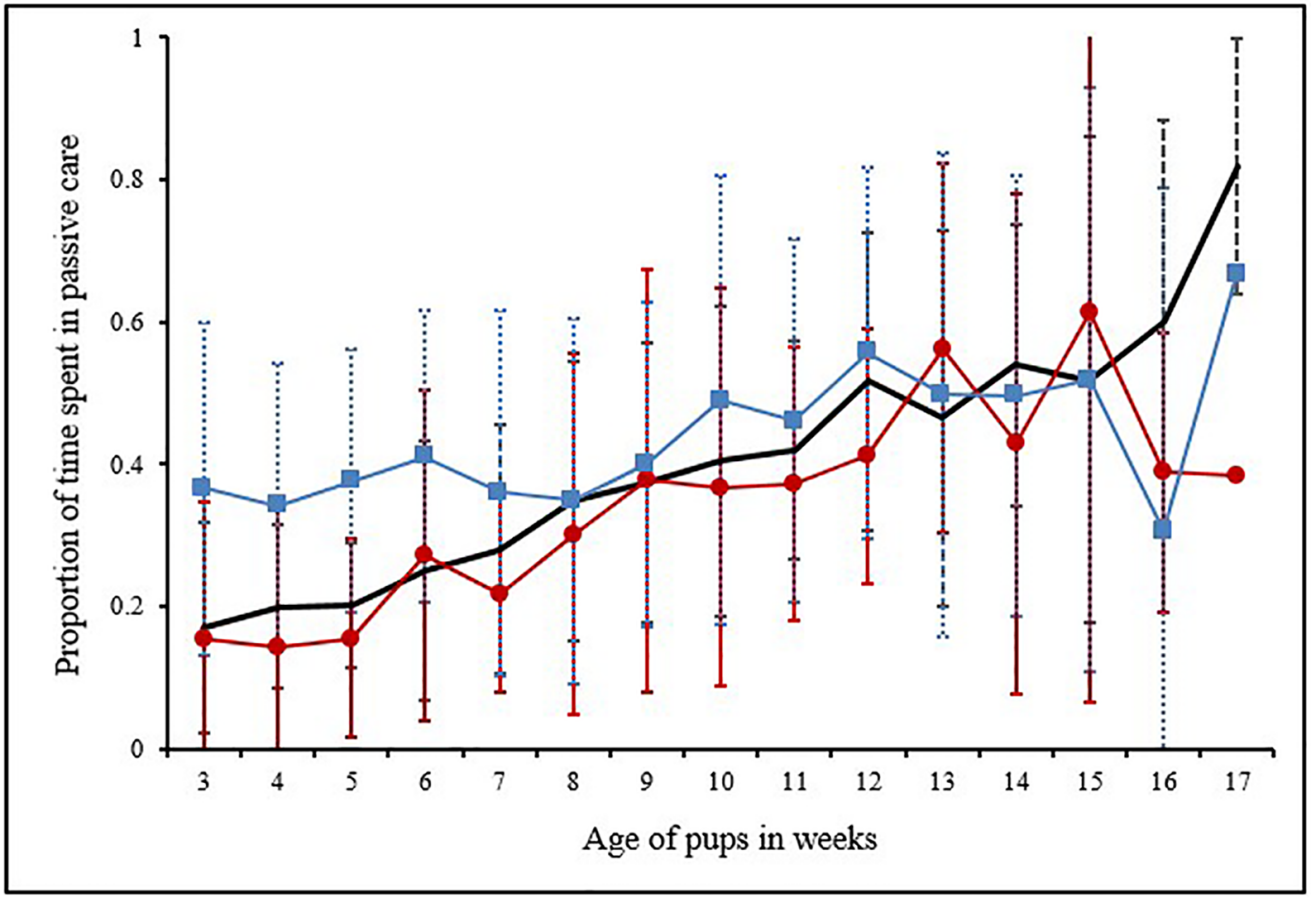
Graphical representation for proportion of time spent in passive care. Line graph showing the mean and standard deviation of the proportion of time spent in passive care by the MO (n = 22), PF (n = 14) and AM (n = 14). The thick line designates the MO, thin line with solid squares designates PF and the line with solid circles is for AM.

Passive care shown by the MO (Mean ± SD: 40.73 ± 17.69%) peaked in the 17^th^ week (81.8%). Passive care by PF (43.98 ± 9.81)% was at its highest in the 17^th^ week (66.67%), and that by AM (33.98 ± 13.92)% was the highest at the 15^th^ week (61.25%). Though active care varied across the caregivers, passive care shown by MO, PF and AM were comparable (ANOVA: *F*_*2*,*42*_ = 1.94, *P* = 1.60) (Fig 5). However, similar to active allocare, passive allocare shown by PF and AM depended on pup age only (GLMM in Table 3, S2 Text).

**Table 3:** The results of the GLMM for passive cares shown by the PF and AM.

	Value	Std. Error	t-value	p-value
**Putative father (PF)**				
(Intercept)	0.21	0.11	1.87	0.06
age	0.02	0.005	4.45	0.00*
ls	0.004	0.02	0.2	0.82
**Allomother (AM)**				
(Intercept)	0.17	0.1	1.65	0.1
age	0.03	0.005	4.74	0.00*
ls	-0.03	0.02	-1.56	0.12

The results of the GLMM considering the proportion of time spent in passive care by the PF and AM as the response variable. Pup age (age) in weeks and litter size (ls) were incorporated as the fixed effects. Litter size ranged from 2 to 6, and the age of the pups ranged from 3 to 17 weeks. The identities of 14 litters (gr) separately for PF and AM and the year of data collection (yr) were the “random effects”.

(*) depicts a significant effect.

### (iii) Grandmother’s care

**Fig 6:**
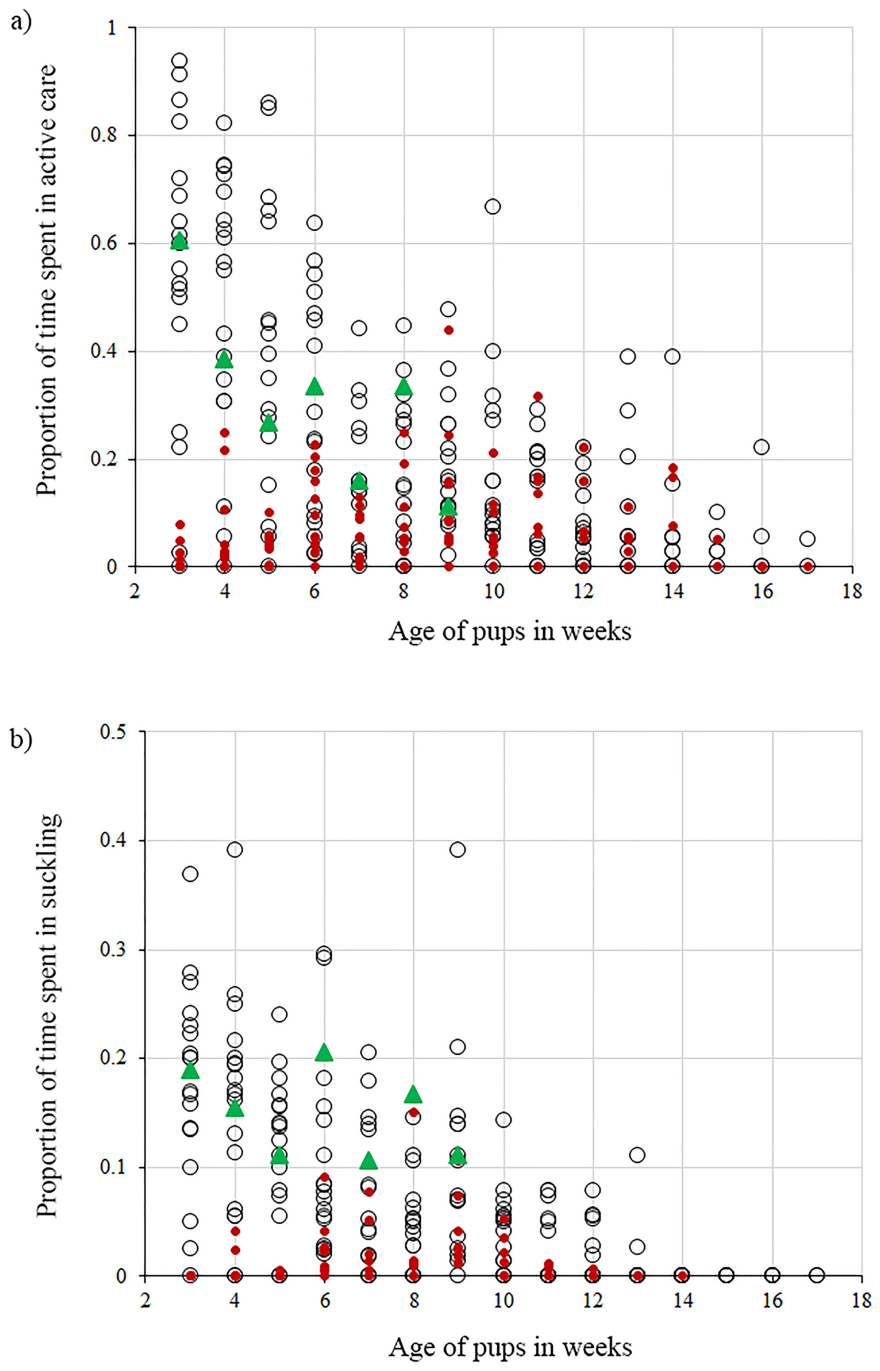
Maternal care vs grandmother’s care. Scatterplots showing the similarity between the MO and PF_2_ grandmother over active care and nursing. Each dot represents a litter where the care has been recorded. a) MO (empty circles) and PF_2_ grandmother (green triangles) spent comparable amounts of their time in active care unlike the AM (red circles). b) Proportion of time spent by PF_2_ grandmother (green triangles) in nursing is comparable with the MO (empty circles) but not with the AM (red circles).

The PF_2_ grandmother provided active care at a level comparable with active maternal care but not with active female-allocare (ANOVA: *F*_*2*,*18*_ = 8.42, *P = 0*.*003*; Post hoc Tukey test: *P* = MO vs PF_2_: 0.99, AM vs PF_2_: 0.007). The nursing effort shown by the grandmother too was comparable to that of the MO (ANOVA: *F*_*2*,*18*_ = 24.83, *P< 0*.*001*; Post hoc Tukey test: *P* = MO vs PF_2_: 0.15, AM vs PF_2_: 0.0001) (Fig 6).

## Conclusions

We observed care by both males and females in social groups of free-ranging dogs, but such care, though quite common, was not ubiquitous. As expected, maternal care was indeed the predominant form of care received by pups [29]. Allocare by both males and females was equally common and contrary to our expectations, included both active and passive forms of care. Though allocare by females was observed at a significantly lower level than maternal care, the overall levels of care provided by the putative fathers was surprisingly comparable to that of the mothers, and much higher than that provided by the allomothers. However, mothers and putative fathers differed in the nature of care that they provided to the pups. Mothers invested a substantial portion of their time activity budgets in energy demanding behaviours like nursing, piling up with pups, allogrooming, etc. that are crucial for the pups’ early stage development and survival. The putative fathers invested most of their effort in play and protection—thus mothers feed and putative fathers play, thereby showing division of responsibilities in pup rearing. Social play has a vital role in the juveniles’ behavioural development [42] and putative fathers seem to contribute significantly to this process.

Female-allocare, although not comparable to the levels of maternal care or male care, allows the pups to gain some extra benefits, which they try to maximize through milk-theft [35]. In spite of being unwilling to nurse the pups [35], the allomothers voluntarily performed behaviours like allogrooming, play, pile sleep and protection towards the non-filial pups, independent of their litter size but not of their age. Pups received allocare as they started to come out of their natal dens, facilitating the increased contact with adult dogs other than their mothers. This also provided pups with ample opportunities for milk-theft from the allomothers, thereby creating a situation of potential conflict for the pups and their allomothers. Interestingly, all the cases of female-allocare observed were between related individuals; aunts, older sisters and grandmothers showed allocare to pups. Hence, while female-allocare could impose a cost on the allomothers, philopatry could reduce the cost by providing inclusive fitness benefits to females that provided care to related pups. In a population that faces high early life mortality [43], any additional care received by pups could help to increase their survival probability, thus making allocaring a stable strategy in such a species. The case study of the grandmother that “adopted” her orphaned grandpups provides support to this idea. She not only adopted and cared for them but she voluntarily nursed like a mother. She had the option of budgeting her time to invest less in nursing and more in other active care behaviours, but she did not.

Allocare by adults is observed in cooperatively breeding species, where adults provide care to non-filial offspring even at an expense to their own reproduction [5,9,44]. In communally breeding species, allocare can provide mutual benefits to females that give birth in each other’s vicinity, and such mutualistic relationships have been speculated to play a role in the evolution of cohesive social systems [20]. Free-ranging dogs live in social groups and have a promiscuous mating system [24,28], which allows for mingling between groups during the mating season. Aggression within the sexes increases during the mating season, but clear reproductive asymmetries, with only some individuals in a group mating, are not evident [45,46]. Individuals often disperse out of their natal groups during the mating season [46], leading to group fission. Unlike cooperatively breeding canids like wolves and coyotes, the dogs don’t usually hunt, and they tend to forage alone most often [24]. Hence social cohesiveness in the dogs is not driven by the inherent need for efficient hunting, as in other social canids [47]. On the contrary, scavenging as a foraging strategy is most efficient for solitary foragers [31,48]. Nevertheless, we observed allocare in 82% of the total observed groups. While we could not be completely sure of the relatedness of the allocaring males with the pups, we knew them to have mated with the mothers of the pups they cared for. All the allocaring females were related to the pups receiving care. We have provided the relatedness estimates between the allomothers and pups considering random mating with unrelated males, but the actual relatedness values are likely to be much higher, considering the promiscuous, multiple mating system of dogs which allows mating between close kin. In fact, in many of the groups the males were indeed brothers of the females, and thus related to their pups. Thus related females denning in close proximity provided allomaternal care to pups, and this lends support to the benefits-of-philopatry hypothesis [49] and to the theory of assured fitness returns, originally proposed to explain the evolution of social behaviour in insects [50]. Further genetic studies, especially to estimate relatedness values with allocaring males could provide further insights into the evolutionary dynamics of alloparental care in the dogs.

Among canids, dogs are the only species in which pups can be raised with minimal parental care, due to help from humans. Earlier studies with pets have mostly reported maternal care, with fathers occasionally defending pups, but not providing any other form of care [51,52]. However, a study on free-ranging dogs in India has reported paternal care, though without direct genetic evidence [29], and our study corroborates this finding. The lack of intraspecific cooperation and cohesiveness in dogs has been suggested to be an effect of domestication, which has led dogs away from a hunting lifestyle to one dependent on stable human generated resources [33,53,54]. Though there have been reports of free-ranging dogs forming large packs to hunt livestock and wildlife in rural and remote areas [32,33], free-ranging dogs in India depend largely on human-generated resources like garbage for their sustenance, but these resources are not necessarily stable, and competition among the scavengers is high.

Our study suggests a highly flexible nature of the breeding system in free-ranging dogs, where maternal care alone can be sufficient for the survival and development of pups, but allocare provides additional benefits to the pups. Male care is relatively easily explained if the care giving males are fathers of the pups. In a promiscuously breeding species that is also philopatric, the inclusive fitness benefits can intensify for males if they breed with related females. In a competitive environment with irregular resources [31] and high early life mortality [43], providing care to non-filial pups is costly, but in the face of high mortality, such care can assure fitness benefits [50] for females in a given breeding season, thereby compensating for the cost of providing care, even when care is snatched in the form of milk-theft [35]. Allocare can also help to build social cohesiveness, increase cooperation within the group, and provide future reproductive benefits to the caregiver [3]. It is interesting to note that such cooperation can arise even in the absence of kin selection. The benefit that could be accrued through inclusive fitness might not be enough to drive a strategy of cooperation among females over pup rearing. On the other hand, increased security of pups from predators [including humans], the possibility of reusing attractive denning sites and a predisposition towards cooperation emanating from the evolutionary history of cooperative hunting might act as proximate causes driving such cooperation.

The free-ranging dogs are more similar to communal breeders like lemurs [7] than their cooperatively breeding relatives, the gray wolves [5]. The high degree of plasticity in the breeding biology of free-ranging dogs leads us to speculate if this could have been one of the adaptations that aided the process of domestication, rather than being a by-product of the process. With a wide spectrum of possibilities including single mothers, caring female relatives, non-caring male and female group members, caring males and even adoptions, the free-ranging dog breeding biology shows the great behavioural plasticity of this species, and provides an interesting model system for understanding the forces that drive the evolution of cooperation.

### Limitations of the study

Though we knew the relationships between the allomothers and the pups receiving care from them, we were unable to judge the relationship between the care-giving males and the pups being cared for. Hence, we could not use genetic relatedness as a variable in our GLMM analyses and could only discuss the possible role of kinship within the groups from a theoretical perspective. Future studies substantiated by genetic data would be required for a deeper understanding of the social dynamics of the dog groups and the evolutionary implications of alloparental care.

## Supporting information

S1 Fig Venn diagram. Venn diagram showing the number of mother-litter units that received maternal care, male care and female allocare. Light red, blue and dark red represents the litters which received maternal care, male care and female allocare respectively.
(TIF)

S1 Table Ethogram representing active and passive care behaviours. A table showing the ethogram for care (modified from Paul et al. 2017). A unique two letter code was used for recording each behaviour during observations. The table shows the code, the name of the behaviour and its description. Maternal/ Allo care was divided into active and passive care. Active care behaviours typically involved direct social interactions between the caregivers and pups. The passive care behaviours were not interactions, but individual actions of the caregiver occurring in the vicinity of the pups which allowed them to share time and space with and provide protection to the pups. In case of social interactions, the individual starting the interaction was designated as the initiator and the one towards which the behaviour was shown, was the recipient (Please see Martin and Bateson 2007 *Measuring Behaviour*: *An Introductory Guide*, 3^rd^ edn. Cambridge University Press). (*) see Bhadra et al. 2016 for different types of food eaten by free-ranging dogs.
(DOCX)

S2 Table Tabulated details of quadratic regression. Table showing the details of quadratic regression. Formula used: lm(formula = femaleallocare ~ week + week^2^). Proportion of time spent in active care by the allomothers (femaleallocare) was considered as the response variable for the quadratic regression. The two variables used are the pup age in weeks (week) and its square (week^2^).
(DOCX)

S3 Table Table showing the AIC values for different data sets. Since we have used the “proportion of time spent in active care” and “proportion of time spent in passive care” as the response variables, we need to find a best fit distribution for the data. We have used “fitdistrplus” package to check the best fit distribution of the data set that are continuous. Proportion of time spent in active care by PF- acarepf, Proportion of time spent in active care by AM- acaream, Proportion of time spent in passive care by PF- pcarepf, Proportion of time spent in passive care by AM- pcaream, PF- Putative father who provided male care to the focal pups, AM- Allomother who provided female-allocare to the focal pups.
(DOCX)

S1 Text Detailed summary of the GLMMs for active care. Text represents the detail summary of the GLMMs for active care shown by the putative fathers (PF) and allomothers (AM) towards focal pups during the observations.
(TXT)

S2 Text Detailed summary of the GLMMs for passive care. Text represents the detail summary of the GLMMs for passive care shown by the putative fathers (PF) and allomothers (AM) towards focal pups during the observations.
(TXT)
