# Supplementary figures and images for: The great Indian joint families of free-ranging dogs
Source: PLoS One. 2018 May 17;13(5):e0197328. doi: 10.1371/journal.pone.0197328 (PMC5957358; doi:10.1371/journal.pone.0197328)

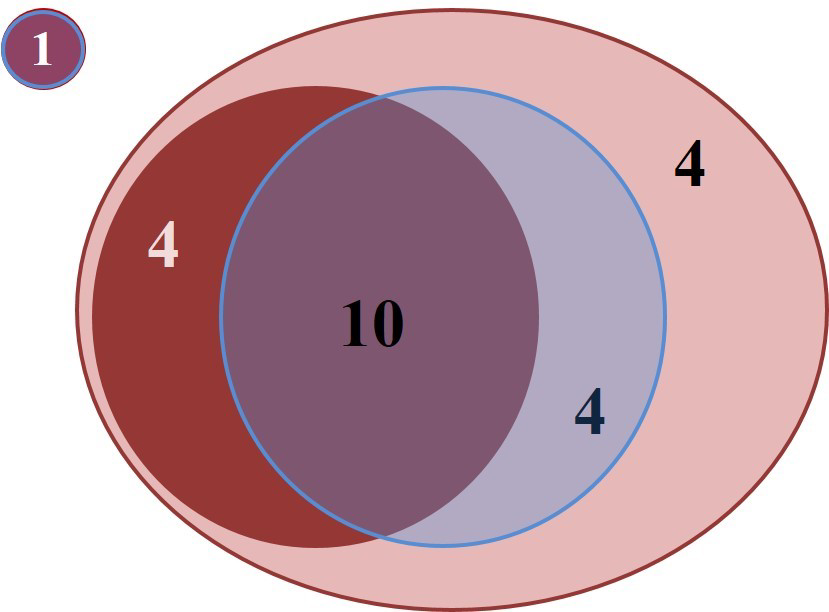

Supplement: S1 Fig — Venn diagram showing the number of mother-litter units that received maternal care, male care and female allocare. Light red, blue and dark red represents the litters which received maternal care, male care and female allocare respectively. (TIF) [file pone.0197328.s001.tif]
