# Supplementary material for: The great Indian joint families of free-ranging dogs
Source: PLoS One. 2018 May 17;13(5):e0197328. doi: 10.1371/journal.pone.0197328 (PMC5957358; doi:10.1371/journal.pone.0197328)
Supplement: S1 Table — A table showing the ethogram for care (modified from Paul et al. 2017). A unique two letter code was used for recording each behaviour during observations. The table shows the code, the name of the behaviour and its description. Maternal/ Allo care was divided into active and passive care. Active care behaviours typically involved direct social interactions between the caregivers and pups. The passive care behaviours were not interactions, but individual actions of the caregiver occurring in the vicinity of the pups which allowed them to share time and space with and provide protection to the pups. In case of social interactions, the individual starting the interaction was designated as the initiator and the one towards which the behaviour was shown, was the recipient (Please see Martin and Bateson 2007 Measuring Behaviour: An Introductory Guide, 3rd edn. Cambridge University Press). (*) see Bhadra et al. 2016 for different types of food eaten by free-ranging dogs. (DOCX) [file pone.0197328.s002.docx]

**The great Indian joint families of free-ranging dogs**

**Manabi Paul^a^ and Anindita Bhadra^a,1^**

^a^Behaviour and Ecology Lab, Department of Biological Sciences, Indian Institute of Science Education and Research Kolkata, India

^1^Behaviour and Ecology Lab, Department of Biological Sciences,

# Indian Institute of Science Education and Research Kolkata

# Mohanpur Campus, Mohanpur,

# PIN 741246, West Bengal, INDIA

*tel.* 91-33-66340000-1223

*fax* **+**91-33-25873020

# *e-mail:* [abhadra@iiserkol.ac.in](mailto:ragh@ces.iisc.ernet.in)

| **Active care** | | |
| --- | --- | --- |
| **Code** | **Behaviour** | **Description of the behaviour** |
| AG | Allo groom | To clean the coat of their pups by licking, to remove dirt and parasites. |
| CK | Nursing | To lie down or stand still in order to allow pups to suckle (feed on breast milk). |
| EF | Eat fecal matter | Mother eats fecal matter of her pups to clean her den. |
| OF | Offer food | Mother brings food obtained through scavenging/ begging in her mouth and drops it on the ground in the vicinity of the pups, allowing them to eat. |
| PL | Play | Individuals engage in affiliative activities like bowing down, tail wagging, mock biting, jumping, tumbling, etc. for enjoyment. |
| PS | Pile sleep | Individuals pile up together while sleeping ensuring maximum body contact. |
| PU | Pile Up | Individuals pile up together while resting ensuring maximum body contact. |
| TH | Threat | Individuals assume an aggressive posture with raised tail and ears, exposed jaws, fore legs stretched forward, producing a deep throated growl and staring at the recipient for a prolonged time. When used to protect pups/juveniles from conspecifics/ other animals, it is considered as an act of care. |
| VM | Vomit | Expels the semi digested and semi solid contents of stomach by regurgitation. |
| **Passive care** | | |
| **Code** | **Behaviour** | **Description of the behaviour** |
| DW  ET  FS  GR  LG  LI  OT | Drink water  Eating food  Food search  Groom  Scratching by legs  Licking  Resting/ standing/ lazing | Individuals drinking water from sources like puddles, shallow drains, dripping taps, etc.  Individuals feeding on any solid or semi-solid food items.  Individuals wander around and search thoroughly (by using the visual, olfactory or tactile cues) for food*.  Individuals clean their own bodies by licking, to remove dirt and parasites.  Individuals use their claws while sitting or standing, to scratch themselves. This is a self-maintenance behaviour.  Individuals pass their tongue over their body to make it clean.  Individuals sit, stand or lie down and remain immobile for several (at least 2) minutes at a stretch. |
