# Supplementary material for: The great Indian joint families of free-ranging dogs
Source: PLoS One. 2018 May 17;13(5):e0197328. doi: 10.1371/journal.pone.0197328 (PMC5957358; doi:10.1371/journal.pone.0197328)
Supplement: S2 Table — Table showing the details of quadratic regression. Formula used: lm(formula = femaleallocare ~ week + week2). Proportion of time spent in active care by the allomothers (femaleallocare) was considered as the response variable for the quadratic regression. The two variables used are the pup age in weeks (week) and its square (week2). (DOCX) [file pone.0197328.s003.docx]

**The great Indian joint families of free-ranging dogs**

**Manabi Paul^a^ and Anindita Bhadra^a,1^**

^a^Behaviour and Ecology Lab, Department of Biological Sciences, Indian Institute of Science Education and Research Kolkata, India

^1^Behaviour and Ecology Lab, Department of Biological Sciences,

# Indian Institute of Science Education and Research Kolkata

# Mohanpur Campus, Mohanpur,

# PIN 741246, West Bengal, INDIA

*tel.* 91-33-66340000-1223

*fax* **+**91-33-25873020

# *e-mail:* [abhadra@iiserkol.ac.in](mailto:ragh@ces.iisc.ernet.in)

**Residuals: Min 1Q Median 3Q Max**

-0.04 -0.02 0.004 0.02 0.04

**Coefficients:**

**Estimate Std. Error t value Pr(>|t|)**

**(Intercept)** -0.09 0.04 -2.44 0.03 *

**week**  0.04 0.008 4.78 0.0004***

**week^2^**  -0.002 0.0004 -5.02 0.0003 ***

Significant codes: 0 ‘***’ 0.001 ‘**’ 0.01 ‘*’ 0.05 ‘.’ 0.1 ‘ ’ 1

Residual standard error: 0.03 on 12 degrees of freedom

Multiple R-squared: 0.68, Adjusted R-squared: 0.63,

F-statistic: 12.88 on 2 and 12 DF, p-value: 0.001
