# Supplementary material for: The great Indian joint families of free-ranging dogs
Source: PLoS One. 2018 May 17;13(5):e0197328. doi: 10.1371/journal.pone.0197328 (PMC5957358; doi:10.1371/journal.pone.0197328)
Supplement: S3 Table — Since we have used the “proportion of time spent in active care” and “proportion of time spent in passive care” as the response variables, we need to find a best fit distribution for the data. We have used “fitdistrplus” package to check the best fit distribution of the data set that are continuous. Proportion of time spent in active care by PF- acarepf, Proportion of time spent in active care by AM- acaream, Proportion of time spent in passive care by PF- pcarepf, Proportion of time spent in passive care by AM- pcaream, PF- Putative father who provided male care to the focal pups, AM- Allomother who provided female-allocare to the focal pups. (DOCX) [file pone.0197328.s004.docx]

**The great Indian joint families of free-ranging dogs**

**Manabi Paul^a^ and Anindita Bhadra^a,1^**

^a^Behaviour and Ecology Lab, Department of Biological Sciences, Indian Institute of Science Education and Research Kolkata, India

^1^Behaviour and Ecology Lab, Department of Biological Sciences,

# Indian Institute of Science Education and Research Kolkata

# Mohanpur Campus, Mohanpur,

# PIN 741246, West Bengal, INDIA

*tel.* 91-33-66340000-1223

*fax* **+**91-33-25873020

# *e-mail:* [abhadra@iiserkol.ac.in](mailto:ragh@ces.iisc.ernet.in)

| **AIC values for:** | | | | |
| --- | --- | --- | --- | --- |
| **Data set** | **Normal** | **Lognormal** | **Exponential** | **Gamma** |
| acarepf | 152.5 | 316.5 | 315.5 | 314.2 |
| acaream | 299.3 | 409.8 | 410.6 | 408.9 |
| pcarepf | 15.5 | 110.3 | 41.3 | 39.2 |
| pcaream | 3.7 | 33.5 | 62.6 | 61.7 |
